# The effect of rehabilitation education through social media on the quality of life in burn patients: a randomized, controlled, clinical trial

**DOI:** 10.1186/s12911-021-01421-0

**Published:** 2021-02-22

**Authors:** Maryam Rouzfarakh, Kolsoum Deldar, Razieh Froutan, Ali Ahmadabadi, Seyed Reza Mazlom

**Affiliations:** 1 School of Nursing and Midwifery, Mashhad University of Medical Sciences, Mashhad, Iran; 2 School of Paramedicine, Shahroud University of Medical Sciences, Shahroud, Iran; 3 Nursing and Midwifery Care Research Center, Mashhad University of Medical Sciences, Mashhad, Iran; 4 Surgical Oncology Research Centre, Mashhad University of Medical Sciences, Mashhad, Iran; 5 Department of Medical-Surgical Nursing, School of Nursing and Midwifery, Mashhad University of Medical Sciences, Mashhad, Iran

**Keywords:** Education, Burns, Rehabilitation, Quality of life, Social media

## Abstract

**Background:**

Burn is one of the most brutal harms to the human body and mind and its wide-ranging complications have many adverse effects on the patients’ quality of life. The present study was conducted to investigate the effect of rehabilitation education through social media on burn patients’ quality of life.

**Methods:**

The present randomized, controlled, clinical trial was conducted on 60 patients admitted to Imam Reza Hospital Burn Center in the city of Mashhad, Iran, who were randomly assigned to either the intervention or control groups (n = 30 per group). The researcher then created a WhatsApp channel to provide educational content and a WhatsApp group for burns patients to join and get their questions answered. The intervention group patients pursued their post-discharge education through the social media for a month. The control group patients received their discharge education according to the ward’s routine procedures through pamphlets and face-to-face training by the personnel. As the study’s main variable, the Burn Specific Health Scale-Brief was completed by both groups before and 1 and 2 months after the intervention. Data were analyzed using the ANCOVA and repeated-measures ANOVA.

**Results:**

There was no significant differences between the intervention and control groups in terms of the QOL score and any of the domains at baseline. The results indicated the significant effect of the intervention both 1 and 2 months post-intervention on the QOL score and all the domains (*P* < 0.05), except for body image (P_model1_ = .550 and  P_model2_ = .463) and skin sensitivity (P_model1_ = .333 and P_model2_ = .104).

**Conclusion:**

The post-discharge rehabilitation education of burns patients through social media improves their quality of life and can be used as an appropriate educational and follow-up method in different stages of the rehabilitation of burn patients.

***Trial registration no.*:**

IRCT20190622043971N1, 05-10-2019.

## Background

Burns and related accidents are the main cause of mortality, disability, and socioeconomic impoverishment throughout the world [1] and can lead to permanent psychological, physical, and social change and impair the quality of life [2, 3]. An annual of 2.4 million people in the world suffer burns, with 650 thousand requiring treatment [4]. In Iran’s 82-million population, 100–150 thousand people suffer burns every year, many of whom develop disabilities [5]. Evidence suggests that burns can leave the most devastating effect on the patients’ quality of life and impair their physical, psychological, social, and spiritual well-being [6]. When the treatment of burn-induced injuries is delayed or neglected, the victim will face irreversible problems such as contracture, formation of hypertrophic scar tissue, heterotrophic ossification, and ongoing increase in stretching of the soft tissue [7]. Moreover, such accidents obviously reduce the patients’ quality of life for different reasons, including severe pain, deformation, and inability to meet personal daily needs [8, 9]. These victims need long-term treatments, such as restorative surgeries and rehabilitating measures [3, 10].

Rehabilitation is a comprehensive, active, and ongoing process that begins with the victim’s admission and in which members of a multidisciplinary team attempt to restore the patient’s physical, psychological, and social skills in preparation for his return to the society and everyday life [11]. Burns rehabilitation should begin immediately after injury and occasionally be continued for several months or years [12]. As irreplaceable members of this team, nurses are responsible for coordinating between the patients, their families, and the rehabilitation team as well as educating the patients and improving their rehabilitation knowledge [13, 14]. In addition, by the continuous assessment of patients during the stages of rehabilitation, nurses are able to detect the victims’ problems early on and refer them to other members of the rehabilitation and medical team and thus help with the continuation of post-discharge care and the prevention of further burns complications [15, 16]. This feature is rather essential, especially for victims admitted to burns centers from remote areas. Since the results of previous studies have shown that rural victims suffer more severe burns with greater complications and longer hospital stays compared to city dwellers [17–20], this group of victims have a greater need for rehabilitation measures. Meanwhile, they often leave the medical and rehabilitation process half-way through due to their long geographical distance from rehabilitation and medical centers and poor socioeconomic status and thus develop undesirable burns complications, such as deformity and several contractures, after a short while.

The lack of access to consistent and equitable health care is one of the main challenges in meeting communities’ needs. Telemedicine is one of the potential solutions to the problem of inequality in the distribution of health care and for facilitating and reinforcing clinical management, training and research [21]. Using this approach can lead to the continuation of the treatment process from the hospital to the patient’s home. Telemedicine services can be provided in different ways, such as through the phone, webpages, mobile apps, or combination models. Several studies have so far discussed the use of these methods for the diagnosis, treatment or improvement in the quality of life of burns injuries [21–25]. One of the cost-effective, simple, and available methods of providing training and care through telemedicine is the use of mobile-based social networks [26], which can have significant effects on the process of patient care and follow-up due to their high response speed and ease of use. Many studies have noted the positive role of social networks in regulating blood pressure in patients with hypertension, blood glucose control in diabetic patients, boosting the effect of antidepressants, helping quit smoking, management of chronic diseases, and weight control behaviour [27–30]. This approach could also be used to alleviate some of the challenges of burns patients in the post-discharge period, including the lack of access to rehabilitation centers with adequately-skilled and competent workers and the heavy costs of transportation.

Imam Reza Hospital Burns Department in the city of Mashhad is currently the only burns treatment center in the northeast of Iran and the only teaching center for special burns care in the country. As such, it admits burns victims from remote regions and even neighboring countries faced with the same challenges. Nonetheless, no studies have yet been conducted on setting up distant rehabilitation programs for patients discharged from this center. As the first study in this center, the present study was conducted to investigate the effect of distant rehabilitation education through social media on the quality of life of burns victims.

## Methods

### Study design

The present clinical trial (Registered in the Iran Clinical Trial Center under the code IRCT20190622043971N1, on 05-10-2019) was conducted on 60 patients admitted to Imam Reza Hospital Burns Department in the city of Mashhad in Iran. The study started after obtaining permission from the university’s Ethics Committee and informed consent from the eligible candidates. The study inclusion criteria were: The patient’s informed consent to participate in the study, 5–60% burns severity, 2nd to 3rd-degree burns, age 10–65 years, the patient’s or his close caregiver’s access to WhatsApp, reading and writing literacy, and speaking Farsi. The exclusion criteria were: The patient’s lack of motivation to continue cooperation, not performing the recommended exercises, moving to other cities, and exacerbation of psychological problems in the course of the study.

### Sample size

With quality of life as the main variable of the study whose data were obtained from a study by Mohaddes-Ardebili et al. [31] and considering a 95% confidence interval and 80% test power and using STATA-16 (StataCorp, College Station, Texas, USA), the sample size was estimated as 23 per group, which was raised to 30 to take account of a potential sample loss of 30%.

### Randomization

The samples were selected based on the inclusion criteria by convenience sampling and were assigned to the intervention and control groups by block randomization with block sizes of 2. The randomization sequence needed for random allocation was generated by the research statistician in STATA-16. Random allocation was carried out by the principal researcher. For concealment, the statistician provided the principal researcher with the random sequence every time.

### Measures

The data collection tool consisted of the Burn Specific Health Scale-Brief (BSHS-B), which is a standard tool with a high internal consistency, validity and reliability for assessing the quality of life of burns patients [32]. This 40-item scale assesses heat sensitivity, body image, hand function, treatment regimens, work, interpersonal relationships, simple abilities, sexuality, and affect and has a scoring system based on a 5-point Likert Scale (from 0 to 4). The total score and the score of each dimension were calculated after normalization by measuring the mean score of the relevant items. The Cronbach alpha coefficient was calculated as 0.73 to 0.85 for the scale dimensions and 0.89 for the entire scale, which confirms the internal consistency reliability of the dimensions and the total score of the scale.

### Implementation phases

#### Phase one: producing the educational content

The content used was prepared based on the latest educational guidelines and in collaboration with medicine, surgery, and physiotherapy department specialists with an expertise in the field of rehabilitation education for burn victims. The content was in the form of educational texts, photos, and videos based on the general needs of burn victims for physiotherapy of different body parts, massage therapy, pain control, and the use of compression clothing and splints. In preparing the content, attempts were made to make the subject matter clear at the start of the video and to specify the educational objectives, maintain simplicity, and ensure that the subject was properly taught to the participants.

#### Phase two: intervention

Once the initial clinical and demographic details were recorded, all the participants were asked to complete the BSHS-B before discharge (often on their last day in the hospital). Both groups then received routine post-discharge rehabilitation training, which included face-to-face verbal training at the patient’s bedside for 15 min plus providing an educational pamphlet with similar content to the verbal training.

*Intervention group* The researcher-designed educational content was provided to the burn victims of this group by creating a WhatsApp channel called “Post-discharge distant rehabilitation”, and a group was also formed on the same application for these patients to directly talk to the rehabilitation team members as well as other victims in the group to share their experiences. All the patients in the intervention group became members of the above channel and group following a one-hour introductory session for familiarization with WhatsApp. Next, general educational texts, photos, and audio or video files were posted on the channel and to the group. If the patients did not wish to ask about their needs and questions in the group, their questions were answered and the educational content was sent to them privately. The patients were asked to perform their own specific rehabilitation program according to the content sent to them for 30–45 min every day based on their ability and endurance. To ensure that the patients performed the program properly, they were asked to send images or short videos of themselves doing the rehabilitation exercises to the medical team members every other day.

*Control group* Before discharge, the control group patients only verbally received the ward’s routine education plus an educational pamphlet provided by the burns department’s nursing personnel.

#### Phase three: after the intervention

One and two months after the patients had joined the rehabilitation education group and channel and their regular attendance and performance were assessed, they completed the BSHS-B in person when visiting the hospital to change their dressings or visit the doctor. This questionnaire was also completed for the control patients at the same time and within the same interval. The patients in both groups were reminded by phone calls to visit the hospital 2 days before the scheduled date.

### Statistical analyses

A statistical analysis was carried out in SPSS-25 (IBM SPSS Statistics, Armonk, NY, USA). The normality of the numeric variables was checked by the Kolmogorov–Smirnov test. Data were presented using the mean (SD) and median (min. and max.) for the numeric normal and non-normal variables, respectively, and frequency (percent) for the categorical variables. The between-group comparisons of the baseline measurements and demographic variables was performed by the independent t test, Mann–Whitney’s U-test and the Chi-square test where appropriate. The repeated-measures analysis of variance (RMANOVA) was used for the within-group comparisons of the three measurements. In this case, the assumption of sphericity was assessed by Mauchly’s test, and then, to correct any deviation from the assumption, the Greenhouse–Geisser correction was applied. The results were followed up using Sidak’s post-hoc test wherever RMANOVA was significant. To assess the effect of the intervention, an analysis of covariance (ANCOVA) was used by way of two models. Model 1: controlling for the baseline measurements, and model 2: controlling for the baseline measurements and confounders, including age, sex, education, occupation, burn percentage, burn depth, and burn cause. A two-way ANOVA with repeated measures was performed to assess the interaction of the measurements by study group. For the non-normal variables, RMANOVAs and ANCOVAs were performed after a logarithmic transformation. All the analyses were carried out using the per-protocol approach and P-values less than 0.05 were considered significant.

## Results

**Fig. 1:**
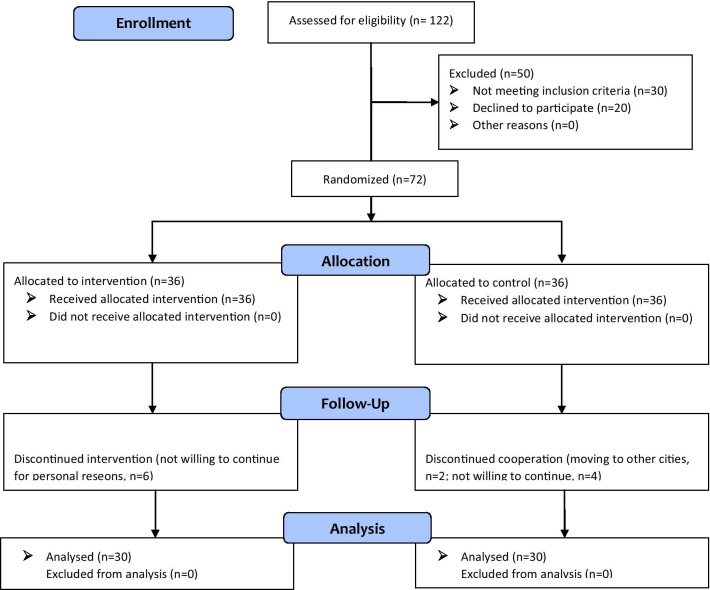
CONSORT flow diagram

One hundred and twenty two patients were recruited for this study. Fifty patients were excluded in the first step, i.e. the eligibility assessment of the candidates (30 did not meet the inclusion criteria and 20 declined to participate). Seventy-two patients were allocated to the intervention (n = 36) and control (n = 36) groups. Six patients from the intervention and six from the control groups discontinued cooperation, making for 60 patients who were ultimately analyzed in the two groups (n = 30 per group) (Fig. 1).

Table 1 presents the patients’ profile. The results showed no significant differences between the intervention and control groups in terms of age, sex, education, burn reason, burn location and burn depth (*P* > 0.05). The most common burn locations were burns of Upper and lower limbs (23.3%) and then Upper limb/Hand (20.0%) in both group.

**Table 1:** Patients’ profile in the intervention (n = 36) and control (n = 36) groups

	Intervention		Control		*P* value^#^
	Mean/median/n	SD/(P25–P75)/%	Mean/median/n	SD/(P25–P75)/%	
Age (year)	32.0	14.3	35.0	12.2	.386
Burn%	20.0	(15.0–25.0)	20.0	(10–35.3)	0.587
Sex (male)	23	76.7%	24	80.0%	1.000
Burn reason					0.754
Heat	29	96.7%	29	96.7%	
Chemical	1	3.3%	1	3.3%	
Burn depth					0.891
II	4	13.3%	2	6.7%	
III	3	10.0%	3	10.0%	
II/III	23	76.7%	25	83.3%	

^#^*P *values were computed using independent t-, Mann–Whitney U- and Chi-square tests where appropriate

### Baseline comparisons of the QOL score and the domains

The results showed no significant differences between the intervention and control groups in terms of the QOL score and any of the domains (*P* > 0.05), indicating the similarity of the groups at baseline with regard to the primary outcomes of the study (Table 2).

**Table 2:** Comparing quality of life score and the domains between intervention (n = 36) and control (n = 36) groups

Variables	Intervention (n = 30)			Control (n = 30)			*P* value^#^	*P* value^###^	*P* value for interaction
	Median	P25	P75	Median	P25	P75			
*Ability to do simple tasks*									
Before intervention	3.0	3.0	5.0	3.0	3.0	5.0	.964^#^	–	**.002**
1-m after intervention	11.0	10.0	12.0	9.0	6.0	9.0	< .001^##^	< .001	
2-m after intervention	12.5	12.0	15.0	9.0	9.0	12.0	< .001^##^	< .001	
*P* value^$^	< .001			< .001					
*Hand functioning*									
Before intervention	5.0	5.0	6.3	5.0	5.0	9.3	.310^#^	–	**.002**
1-m after intervention	18.0	14.8	21.0	15.0	10.0	16.0	.002^##^	.006	
2-m after intervention	20.0	20.0	23.5	15.0	13.8	17.3	< .001^##^	< .001	
*P* value^$^	< .001			< .001					
*Emotional*									
Before intervention	16.0	13.0	24.3	16.5	13.0	21.8	.894^#^	–	**.021**
1-m after intervention	34.0	31.8	35.0	24.5	21.0	28.3	< .001^##^	< .001	
2-m after intervention	35.0	32.0	35.0	27.0	24.0	28.0	< .001^##^	< .001	
*P* value^$^	< .001			< .001					
*Body image*									
Before intervention	8.0	4.0	11.3	8.0	6.0	9.3	.976^#^	–	**< .001**
1-m after intervention	10.0	8.0	13.0	12.0	8.0	14.3	.550^##^	.463	
2-m after intervention	16.0	15.8	20.0	13.0	10.8	16.0	< .001^##^	< .001	
*P* value^$^	< .001			< .001					
*Inter-personal relations*									
Before intervention	13.5	8.0	18.0	11.5	8.0	16.0	.243^#^	–	.094
1-m after intervention	19.0	17.0	20.0	15.5	12.0	20.0	< .001^##^	.001	
2-m after intervention	20.0	16.0	20.0	14.0	9.8	16.0	< .001^##^	< .001	
*P* value^$^	< .001			.013					
*Sexual functioning*									
Before intervention	6.0	3.0	9.8	3.5	3.0	8.0	.134^#^	–	.591
1-m after intervention	13.0	10.8	15.0	9.0	6.8	15.0	.007^##^	.008	
2-m after intervention	14.5	12.0	15.0	11.5	9.0	14.0	.001^##^	.006	
*P* value^$^	< .001			< .001					
*Skin sensitivity*									
Before intervention	5.0	5.0	7.8	5.0	5.0	7.3	.952^#^	–	**< .001**
1-m after intervention	11.0	9.0	15.3	15.0	10.0	18.0	.333^##^	.104	
2-m after intervention	17.0	16.0	19.3	12.0	10.0	14.3	< .001^##^	< .001	
*P* value^$^	< .001			< .001					
*Care quality*									
Before intervention	9.0	6.8	10.3	8.5	5.0	10.0	.384^#^	–	**.004**
1-m after intervention	19.0	17.0	21.0	13.0	10.0	15.0	< .001^##^	< .001	
2-m after intervention	20.0	20.0	21.3	15.0	13.5	18.5	< .001^##^	< .001	
*P* value^$^	< .001			< .001					
*Occupation*									
Before intervention	4.0	4.0	4.3	4.0	4.0	4.0	.894^#^	–	**< .001**
1-m after intervention	15.0	8.8	17.0	8.0	4.8	12.0	< .001^##^	< .001	
2-m after intervention	16.0	15.5	20.0	12.0	8.0	12.0	.068^##^	< .001	
*P* value^$^	< .001			< .001					
*QOL score*									
Before intervention	76.3	17.9		74.9	17.4		.760^#^	–	**< .001**
1-m after intervention	147.6	14.2		116.0	21.6		< .001^##^	< .001	
2-m after intervention	172.0	8.1		129.0	21.5		< .001^##^	< .001	
*P* value^$^	< 0.001			< 0.001					

*QOL* Quality of Life, *P* Percentile

Data are expressed as mean (SD) for QOL score and median (P25–P75) for other domains

^$^*P* values for within group comparisons were computed using repeated measures analysis of variance for QOL score and after logarithmic transformation for other domains

^#^*P* values for between group comparisons at baseline were computed using independent t-test for QOL score and Mann–Whitney U-test for other domains

^##^Model1: *P* values for between group comparisons at 1-month and 2-month after intervention were computed using analysis of covariance (ANCOVA) after controlling for baseline measures for QOL score and after logarithmic transformation for other domains

^###^Model2: *P* values for between group comparisons at 1-month and 2-month after intervention were computed using ANCOVA after controlling for baseline measures and confounders (including age, sex, education level, occupation, burn percent, burn depth, and burn cause) for QOL score and after logarithmic transformation for other domains

### Within-group comparisons of the QOL score and the domains

Table 2 presents the results of the within-group comparisons of the QOL score and the domains in each group. There was a significant time effect for the QOL score and all the domains within both groups (*P* < 0.05). In addition, the results of Sidak’s post-hoc test showed significant differences between the 1-month and 2-month interventions compared to the baseline measurements in both groups for the QOL score and all the domains. Nonetheless, the results of Sidak’s test showed no significant differences between the 1-month and 2-month follow-up measurements in terms of affect, interpersonal relationships, and sexuality in either of the two groups; in addition, no significant differences were observed between the 1-month and 2-month follow-up measurements in terms of hand function, body image, heat sensitivity and QOL score in the control group (*P* > 0.05).

### The results of the interaction between measurements and groups

**Fig. 2:**
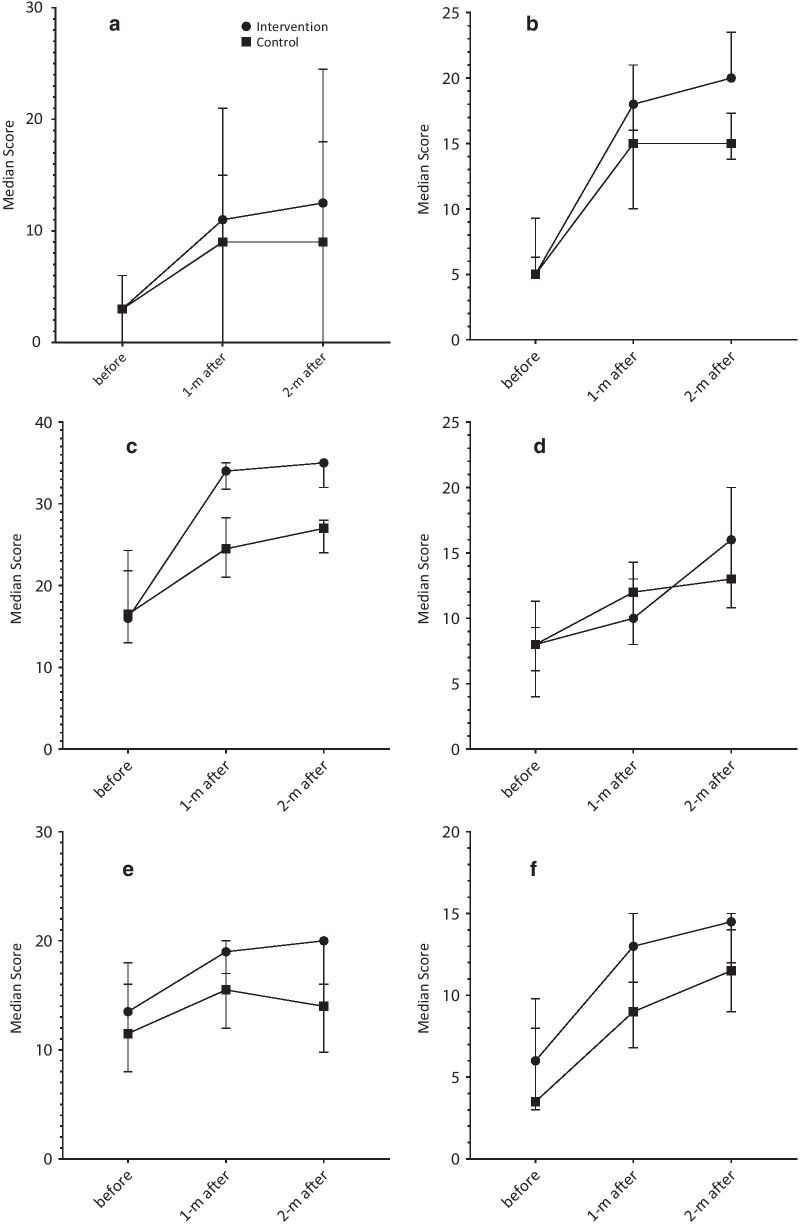

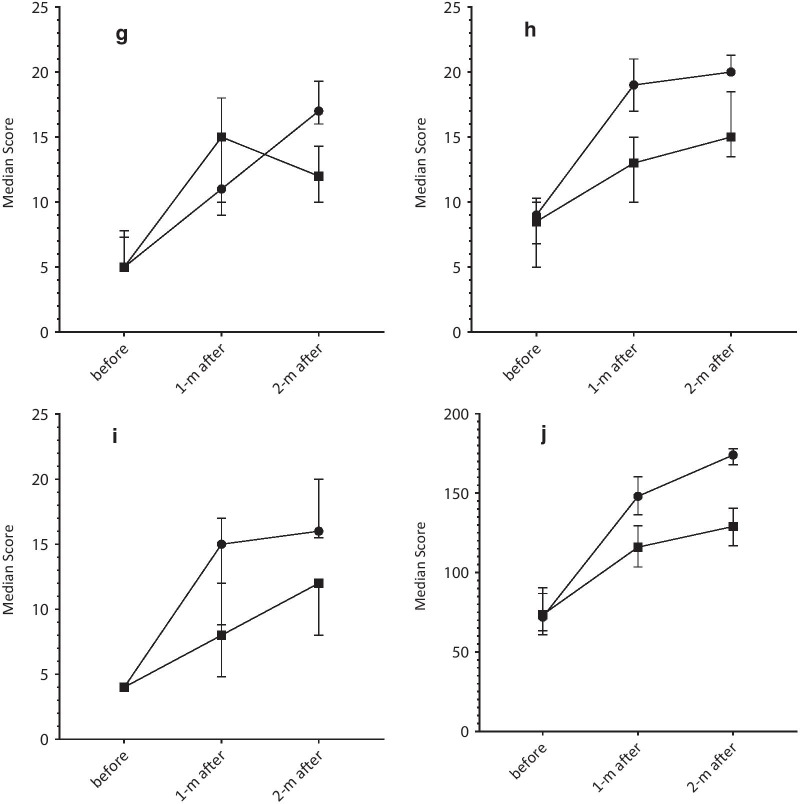
Measurements of quality of life score and the domains in baseline, 1-month and 2-month after intervention in the intervention (n = 36) and control (n = 36) groups. **a** Ability to do simple tasks; **b** hand functioning; **c** emotional; **d** body image; **e** inter-personal relations; **f** sexual functioning; **g** skin sensitivity; **h** care quality; **i** occupation; **j** QOL score

Table 2 and Fig. 2 present the results of the interaction between measurements and groups. Significant interactions were observed between measurements and groups for the QOL score and all the domains (*P* < 0.05), indicating the significantly different time trend of measurements between the intervention and control groups. The exceptions were interpersonal relationships (*P* = 0.094) and sexuality (*P* = 0.591) domains.

### The intervention effect

Table 2 presents the results of the ANCOVA by the two models. In both models, the results showed a significant intervention effect for both the 1-month and 2-month follow-up measurements in the QOL score and all the domains (*P* < 0.05), except for body image (P_model1_ = 0.550 and P_model2_ = 0.463) and skin sensitivity (P_model1_ = 0.333 and P_model2_ = 0.104).

## Discussion

The results of the present study conducted to investigate the effect of rehabilitation education through social media on quality of life in burns patients showed an improvement in the mean scores of simple abilities, hand function, affect, body image, interpersonal relationships, sexuality, heat sensitivity, treatment regimens, and work in the burns patients in both the intervention and control groups 1 and 3 months after the intervention, but this improvement was significantly higher in the intervention group compared to the controls. In other words, after implementing the rehabilitation program through the social media, the scores increased in both groups in all the dimensions of health-related QOL measured by the BSHS-B, but this increase was significantly higher in the intervention group compared to the controls.

These results agree with those of previous studies that had sought to improve the QOL of burns victims through various interventions such as taking part in meetings [33, 34], distant education and follow-up on the phone [35–37], or using multimedia educational CDs [31, 38]. In a study conducted by Heydarikhayat et al. (2018), in addition to home visits, the patients were followed-up on the phone, and the mean scores of simple abilities, body image, treatment regimens, and work one and half months after discharge were significantly higher in the intervention group compared to the controls [36]. In the cited study, exercising the affected joints, skin care before the exercises, scar management, and wearing compression clothing were among the most important items assessed during the medical team’s interaction with the victims. A similar phone intervention was used in the study by Rezaei et al. [37], and their results showed a significant increase in the mean scores of hand function, body image, interpersonal relationships, heat sensitivity, work and sexuality in the tele-nursing (phone contact) group after the intervention compared to the control and face-to-face education groups [37]. These interventions seem to have rapidly improved the appearance of burn wounds due to the active participation of the patients in self-care, since complications such as wound infection were not reported in the intervention group victims. The telephone follow-up of burn victims reduced symptoms such as anxiety and posttraumatic stress as well as emotional problems and improved the health status [35].

The effectiveness of educational self-care videos on the quality of life of burn victims has also been confirmed in a study conducted by Ardebili et al. [31]. Their results showed that using multimedia self-care education improves the patients’ quality of life score, especially in the physical dimension, by even more than twice compared to patients receiving routine education. A similar intervention was also reported effective in reducing the anxiety of burns victims [38]. The limitations of the noted methods include being offline, which means that the patients were unable to have a two-way communication with their care team, and no strategy had been devised in them to respond to the patients’ possible problems or questions.

WhatsApp is one of the applications installed on smartphones that is widely available to the public, and its use in various studies has suggested improved communication between different levels of burns care providers and other medical personnel, reduced costs of hospital visits, and reduced unnecessary visits to specialized centers [21, 22]. Many studies have noted the positive role of social networks in moderating the blood pressure of patients with hypertension, blood glucose control in diabetic patients, increased effect of antidepressant therapies, helping people quit smoking, and the management of chronic diseases [27–29]. Nonetheless, no study was found on the use of this or similar applications in the distant rehabilitation of burn victims.

The results of previous studies suggest that post-discharge rehabilitation programs improve the physical, social, and psychological functioning of patients after burns [39]. Given the effective role of an ongoing care model in improving the patients’ QOL [40], using a social networking application in the present study improved the patients’ QOL through a two-way and ongoing communication between the victims and the medical team and the exchange of information needed to manage post-discharge rehabilitation. The continuous post-discharge interaction of the medical team with the patients through similar tools makes patients feel continually supported and followed-up by the medical team and they thus more actively take part in rehabilitation programs with greater motivation and readiness, so that they can quickly return to their former levels of functioning pre-injury. Clearly, improved physical condition and medical teams’ support even after discharge can have a significant effect on the patients’ psychological status and ultimately QOL.

Moreover, using interactive tools such as WhatsApp can increase the patients’ performance in the process of rehabilitation. The implementation of educational programs based on burn victims’ needs improves their adherence to the treatment regimen [41]. Therefore, another potential reason for the positive results obtained in the present study may be the rapid responding to the patients’ real needs by the medical team, since patients could quickly contact the medical team members and receive a proper and prompt response if they faced any issues or ambiguities regarding the rehabilitation exercises.

Since there is currently no follow-up and post-discharge system for burn victims in the biggest burns care center of eastern Iran and given the positive results of the present study regarding the use of WhatsApp, this or similar tools can be used as an easily-accessible, inexpensive, and effective means of communication in distant rehabilitation education and follow-up. Since burns rehabilitation care can take place even at the patients’ home in all social strata, using this strategic method can contribute to equity in health care for all people.

## Conclusion

According to the results, the post-discharge follow-up and education of patients using WhatsApp leads to the continuation of the patients’ communication with the nurses, doctors, and medical team and can be proposed as a cost-effective educational and follow-up method in various stages of the rehabilitation of burns patients. Since third- and fourth-degree burns are associated with profound and devastating changes in the patients’ life, the long-term follow-up of burns survivors, such as through social media applications like WhatsApp, is recommended.

### Limitations

The limitations of the present study included the short follow-up period, some victims’ lack of internet access (leading to their exclusion), and the non-assessment of patients with higher degrees of burns (due to their longer treatment duration). Furthermore, given the relatively small sample size of the present study, manually registering the patients’ details and following up with them on WhatsApp was practical. Meanwhile, for larger numbers of victims, using a way of connecting to the hospital HIS should be considered as a better solution. In addition, the users’ images and videos being stored in the medical team members’ phones was a challenge of this study. Therefore, the patients were asked to make their photos and videos unrecognizable, and at times when doing so was not possible, the medical team members were asked to manually delete the patients’ images and videos from their phones at the end of each work day.

## Data Availability

The datasets generated and analyzed during the current study are not publicly available due to their containing information that could compromise the privacy of research participants, but are available from the corresponding author on reasonable request.
